# Chinese EFL learners’ empowerment: does teacher care and confirmation matter?

**DOI:** 10.3389/fpsyg.2023.1273004

**Published:** 2023-09-26

**Authors:** Min Li, Zhesen Chu

**Affiliations:** ^1^Department of Foreign Languages and Economics, Jiaozuo Normal College, Jiaozuo, Henan, China; ^2^School of Physical Education (Main Campus), Zhengzhou University, Zhengzhou, China

**Keywords:** rhetorical/relational goal theory, learner empowerment, teacher care, teacher confirmation, EFL learners, China

## Abstract

**Introduction:**

Inspired by the rhetorical/relational goal theory, the current inquiry evaluated the role of two teacher relational behaviors, namely care and confirmation, in predicting Chinese EFL learners’ empowerment.

**Methods:**

To do this, we administered three closed-ended scales to 391 Chinese students who studying English as a foreign language in various educational institutions in China. Students’ attitudes toward the interplay between teacher care, teacher confirmation, and learner empowerment were statistically analyzed using IBM SPSS Amos software.

**Results:**

The results uncovered that teacher confirmation and teacher care serve an essential role in improving Chinese EFL learners’ empowerment. This indicates that EFL learners’ empowerment strictly depends on the relational behaviors that teachers employ in instructional-learning contexts.

**Discussion:**

The study outcomes may have some theoretical and practical implications for L2 researchers, language teachers, and teacher trainers.

## 1. Introduction

Learner empowerment is a key precursor to affective, cognitive, and behavioral learning (Wei et al., 2021; Derakhshan, 2022; Pan, 2022; Li et al., 2023), and therefore, is an essential dimension of any successful learning experience, including foreign and second language learning. As a motivation-based construct, learner empowerment pertains to a psychological state in which “learners feel competent, find the required course tasks meaningful, and feel like they have an impact on the learning process” (Ledbetter and Finn, 2016, p. 4). As put by Pan (2022), learners who feel empowered typically have favorable attitudes toward the instructor, the classroom environment, and the learning process. Such positive attitudes inspire learners to make the most of the learning opportunities (Dewaele and Proietti Ergün, 2020; Wang and Derakhshan, 2023) and to actively engage in the learning tasks (Ferrer et al., 2020; Pan et al., 2023; Wang et al., 2023). In describing the value of learner empowerment, You (2016) further stated that empowered learners are more inclined to pursue their academic goals as they think what they are doing in classroom contexts is precious.

In accordance with all these, the factors that facilitate or inhibit learner empowerment need to be uncovered. In line with this necessity, some earlier studies (e.g., You, 2016; Davis and Bowles, 2018; Dağgöl, 2020, among others) have evaluated the influence of learner-related factors on learner empowerment. Furthermore, some inquiries (e.g., Yujing, 2015; León and Castro, 2017; Pan, 2022, among others) have assessed the impact of context-related factors on this motivation-based construct. Likewise, a body of research (e.g., Ledbetter and Finn, 2013, 2016; Cakır, 2015; Diaz et al., 2016; Wang et al., 2022; Wang and Hemchua, 2022; Wang and Derakhshan, 2023; Zhi and Wang, 2023) has looked into the effect of teacher-related factors on learner empowerment. Yet, the role of some teacher-related factors, including teacher care and teacher confirmation, in promoting or impeding learner empowerment has remained underexplored. Inspired by this shortcoming, the current paper aims to inspect the function of these teacher-related factors in predicting Chinese EFL learners’ empowerment.

One teacher-related factor that may play some role in increasing or decreasing learner empowerment is teacher care. Teacher care is a positive communication behavior that teachers use to address the psychological, emotional, and academic needs of learners (Noddings, 2012). In the words of Laletas and Reupert (2016), teacher care pertains to “teachers’ behavioral attempts to meet students’ emotional and psychological needs through creating a nourishing, supportive, positive, and respectful classroom climate” (p. 486). This is similarly reflected in the definition of teacher care developed by Derakhshan et al. (2022) who described this concept as the verbal or nonverbal behaviors that instructors employ to demonstrate their understanding of and responsiveness to students’ needs and wants. As pointed out by Dickinson and Kreitmair (2019), this positive communication behavior assists teachers to establish close and amicable relationships with their learners. In the same vein, Song et al. (2022) noted that teachers’ caring behavior enables them to build strong rapport with their pupils, which in turn, promotes students’ academic engagement (Havik and Westergård, 2019; Pan et al., 2023), learning motivation (Liu and Chiang, 2019), and psychological well-being (Lan and Moscardino, 2019; Pan et al., 2023).

Another teacher-related factor that may make a noticeable change in learner empowerment is teacher confirmation. Teacher confirmation is another instance of positive communication behavior by which teachers inspire their learners and make them feel valued (Goldman and Goodboy, 2014). According to Ellis (2004), teacher confirmation is the process “that includes actions on the part of teachers that cause learners to feel endorsed, recognized and acknowledged” (p. 2). As put by Goldman et al. (2018), the confirming actions that learners receive in the learning environment can drastically influence their thoughts, feelings, and perceptions about the educational process. Moreover, as Hsu and Huang (2017) mentioned, the confirming cues that teachers typically offer in educational environments tremendously decrease learners’ classroom apprehension, as a result of which learners’ academic motivation (Croucher et al., 2021) and willingness to attend classes (Wang and Derakhshan, 2023) might be increased.

In view of the key role of teacher care and teacher confirmation in promoting learners’ academic engagement, motivation, and well-being, many scholars (e.g., Peaslee, 2018; Ramberg et al., 2019; Johnson and LaBelle, 2020; Lavy and Naama-Ghanayim, 2020; Wang et al., 2022; Pan et al., 2023; Wang and Derakhshan, 2023, to cite a few) have studied these communication behaviors and their implications in general education contexts. Likewise, some language scholars (e.g., Gabry’s-Barker, 2016; Derakhshan et al., 2019; Gkonou and Miller, 2019; Wang and Derakhshan, 2023, among others) have inspected these interpersonal behaviors and their favorable or unfavorable consequences in second and foreign language classes. Yet, to the best of our knowledge, not sufficient inquiry to date has explored the possible effects of these teacher communication behaviors on language learners’ empowerment. In fact, whether teacher care and teacher confirmation can bring significant changes in language learners’ empowerment is not addressed by earlier studies, which justifies the underlying purposes of the current research.

## 2. Literature review

### 2.1. Theoretical foundation

The interplay between teacher caring, teacher confirmation, and learner empowerment can be interpreted through the “rhetorical/relational goal theory” (Mottet et al., 2006). This theory is mainly centered on “the rhetorical and relational goals that teachers and students have and how these goals guide the instructional communication that is transacted in the classroom” (Mottet et al., 2006, p. 260). This implies that teachers work toward a set of rhetorical and relational goals that may enhance their teaching effectiveness (Myers et al., 2018). According to Myers et al. (2018), rhetorical goals modify the instructional messages teachers use to regulate their learners’ actions, thoughts, and viewpoints. Relational goals, on the other hand, assist teachers in creating and maintaining strong bonds with learners (Myers et al., 2018). To meet the rhetorical objectives, teachers need to employ various communicative behaviors (e.g., humor, clarity) in order to clearly convey the required knowledge and information to their learners (Houser and Hosek, 2018). To achieve the relational objectives, they are required to use different communicative behaviors (e.g., care, confirmation) in order to build amicable and mutual relationships with their learners (Houser and Hosek, 2018). Central to this theory is the idea that learners hold various relational and academic needs, which need to be answered by their teachers. As pointed out by the proponents of this theory (Houser and Hosek, 2018; Myers et al., 2018), fulfilling the academic and relational needs of learners through positive communicative behaviors helps teachers improve their learners’ educational behaviors like interest, engagement, and empowerment.

### 2.2. Teacher care

The term “teacher care” is a multifaceted and ambiguous concept that is open to various explanations (Isenbarger and Zembylas, 2006). For Rogers and Webb (1991), teacher care means showing sensitivity to learners’ desires and abilities, and providing them with appealing tasks and materials. According to Goldstein and Lake (2000), teacher care refers to “the establishment of meaningful relationships, the ability to sustain connections, and the commitment to respond to others with sensitivity and flexibility” (p. 862). Teven (2007) further characterized this construct as the extent to which teachers promptly and decisively respond to students’ demands. Additionally, Noddings (2012) described this concept as any teacher-prompted action improving teacher-student relationships in classroom contexts. Inspired by these interpretations, Gabry’s-Barker (2016) conceptualized this construct in the language education domain as empathizing with language learners, demonstrating interest in their learning, and offering genuine support to them. As put by Teven (2007), teacher care is a multidimensional construct that entail three different components: “*empathy*,” “*understanding*,” and “*responsiveness*.” According to Teven (2007), the first component of teacher care, known as empathy, pertains to “an individual teacher’s ability to identify with his or her learners’ situation or feelings” (p. 382). The second component, which is called understanding, refers to an individual teacher’s capacity to perceive his or her learners’ thoughts, ideas, and demands (Teven, 2007). The third component of this construct, as pointed out by Teven (2007), alludes to “being learner-oriented and having sensitivity toward learners” (p. 383).

Establishing a caring environment not only influences the personal characteristics of learners (Xie and Derakhshan, 2021; Whitehead et al., 2023), but also brings some noticeable changes in their academic achievements (Higareda, 2018; Schat, 2023). In this respect, Lavy and Naama-Ghanayim (2020) posited that teachers’ assertive and immediate reactions to learners’ desires, wants, or problems can tremendously improve learners’ self-esteem and well-being. Maloney and Matthews (2020) articulated that teacher caring behaviors can grow the sense of connectedness in learners. In a similar vein, Zhang (2021) argued that having sensitivity toward learners can lead them to higher levels of vigor, absorption, and dedication, which are three important dimensions of student academic engagement (Carmona-Halty et al., 2019). It stands to reason that the high level of academic engagement paves the way for learners to achieve desired academic outcomes (Lei et al., 2018; Martínez et al., 2019).

The critical role of teacher care in improving educational outcomes has stimulated many scholars (e.g., Gasser et al., 2018; Dickinson and Kreitmair, 2019; Derakhshan et al., 2022; Song et al., 2022, among others) worldwide to study this variable and its beneficial impacts on learners’ classroom behaviors. Dickinson and Kreitmair (2019), for instance, evaluated the role of teacher caring behaviors in students’ classroom efforts. In doing so, a group of undergraduate students were invited to fill out two reliable surveys. The study results indicated that caring behaviors significantly promote students’ efforts and academic achievements. In another inquiry in language education context, Derakhshan et al. (2022) assessed the function of teacher care in fostering English learners’ engagement. To this aim, two self-report questionnaires were distributed among a sample of Iranian and Polish EFL learners. The outcomes of this inquiry revealed that teacher care serves a key role in enhancing English language learners’ engagement. In the same vein, Song et al. (2022) examined the influence of teacher caring on English language learners’ willingness to communicate. For this purpose, a sizable sample of Chinese English learners was asked to cooperate in the data-gathering process. The study findings uncovered that English learners who feel valued and cared for are more inclined to communicate in learning environments.

### 2.3. Teacher confirmation

The term “teacher confirmation” has been broadly characterized by Ellis (2000) as “the transactional process by which teachers talk and interact with students that make them feel they are valuable and significant individuals” (p. 265). In the instructional milieu, this communication behavior mainly implemented within three dimensions of “*responding to questions*,” “*demonstrating interest*,” and “*interactive teaching style*” (Goldman et al., 2018). Responding to questions, as the first dimension, is about the amount of time teachers spend answering their learners’ inquiries (Shen and Croucher, 2018). Demonstrating interest, as the second dimension, alludes to the level of enthusiasm or excitement teachers demonstrate for their learners’ success (Gao, 2021). The last dimension of teacher confirmation, called interactive teaching style, concerns the instructional method teachers choose in accordance with their learners’ academic demands (Johnson and LaBelle, 2023). By taking these affirmative steps, teachers can create strong emotional bonds with their learners and provide them with a relaxed, enjoyable learning atmosphere (Goldman et al., 2014; Burns et al., 2018).

Multitudes of studies to date (e.g., Houser and Waldbuesser, 2017; Hsu and Huang, 2017; Peaslee, 2018; Wang and Derakhshan, 2023, among others) have been conducted on teacher confirmation and its pedagogical implications. For one, Hsu and Huang (2017) inspected the effects of teacher confirming cues on university students’ academic apprehension. In doing so, two self-report instruments were administered to 121 university students. The result exhibited that the confirming cues students receive throughout the learning process help them mitigate their classroom apprehension. In another study, Peaslee (2018) looked into the role of teacher confirmation in improving university students’ self-efficacy. To accomplish this, two pre-designed surveys were given to a group of college students. The findings of this investigation showed that teacher confirmation can strengthen students’ self-efficacy beliefs. In addition, in a cross-cultural inquiry, Wang and Derakhshan (2023) scrutinized the predictive function of teacher confirmation in Iranian and Chinese students’ willingness to attend classes. To achieve this goal, two groups of Iranian and Chinese students were recruited to take part in the research process. The study outcomes divulged that teacher confirmation can dramatically enhance the willingness of Iranian and Chinese students to attend classes.

### 2.4. Learner empowerment

The construct of empowerment has been generally characterized as the humanistic process of “adopting the values and practicing the behaviors of enlightened self-interest so that personal and organizational goals may be aligned in a way that promotes growth, learning, and fulfillment” (Luechauer and Shulman, 1993, p. 14). Referring to this definition, Frymier et al. (1996) particularly described learner empowerment as a mental state in which “a learner finds the tasks meaningful, feels competent to perform them, and feels his/her efforts have an impact on the scheme of things” (p. 183). Central to this description is the idea that learner empowerment is multifaceted construct with three interrelated dimensions, including “*meaningfulness*,” “*competence*,” and “*impact*” (Houser and Frymier, 2009). Meaningfulness pertains to the degree to which an individual learner finds the present course engaging and worthwhile (Finn and Schrodt, 2012). Competence also refers to individual learners’ assessment of their ability to fulfill the course requirements (Ledbetter and Finn, 2013). Finally, impact alludes to an individual learner’s perceptions of his or her influence on the learning process (Cakır, 2015). Feelings of meaningfulness, competence, and impact trigger learners’ academic motivation (Brooks and Young, 2011) and give them extra energy to follow their academic goals (You, 2016).

As put by Houser and Frymier (2009), learner empowerment is the outcome of learners’ individual characteristics and teachers’ communication behaviors. Considering this, a number of investigations (e.g., Finn and Schrodt, 2012; Brunton and Jeffrey, 2014; Diaz et al., 2016; Dağgöl, 2020; Teng and Zhang, 2020; Derakhshan, 2022) have been conducted in both language education and general education settings to locate the learner- (e.g., self-regulated strategies, academic motivation) and teacher-related (e.g., teacher power, teacher clarity, teacher nonverbal immediacy) determinants of learner empowerment. As to the learner-related determinants, Dağgöl (2020), for instance, explored the function of academic motivation in promoting Turkish English learners’ empowerment. To do so, two validated scales were administered to 150 Turkish high school students. The study findings displayed that academic motivation can strengthen the sense of empowerment in EFL learners. In another study, Teng and Zhang (2020) examined the role of self-regulated strategies in empowering L2 learners. To achieve this purpose, two self-report questionnaires were sent to 80 undergraduate students. The analysis of the questionnaires represented that self-regulated strategies play a critical role in enhancing L2 learners’ empowerment.

As to the teacher-related determinants, Finn and Schrodt (2012) investigated the effects of teacher nonverbal immediacy and teacher clarity on undergraduate students’ empowerment. To this aim, three reliable measures of the variables were shared among a sample of undergraduate students. The outcomes of the inquiry evinced that these two communication behaviors positively affect students’ empowerment. In a similar vein, Diaz et al. (2016) studied the influence of teacher power on English learners’ sense of empowerment. To accomplish this, two reliable measures of the variables were distributed among 1,213 undergraduate students. The results uncovered that English learners’ perceptions of teacher power can raise their sense of empowerment. Notwithstanding this body of research, the role of some teacher communication behaviors such as teacher care and teacher confirmation in empowering language learners has remained unknown. To address this lacuna, this inquiry intends to evaluate the role of teacher care and teacher confirmation in fostering Chinese EFL learners’ empowerment. To achieve this aim, this research plans to address the following questions:

- To what extent, if any, is there any significant relationship between teacher care, teacher confirmation, and learner empowerment?
- To what extent, if any, do teacher care and teacher confirmation predict Chinese EFL learners’ empowerment?

## 3. Method

### 3.1. Participants

In the current research, the participants were 391 EFL learners recruited from different educational institutions in China. The participants in this inquiry were chosen based on the convenience sampling strategy. As put by Dörnyei and Csizér (2012), convenience sampling is “a non-probability sampling method through which subjects are typically selected due to their geographical proximity, availability, and easy accessibility” (p. 82). They were 359 females (92%) and 32 males (8%), holding different academic degrees (i.e., bachelor’s degree, master’s degree, and doctoral degree). Their age varied from 20 to 29 years old (*M* = 24.5, *SD* = 3.6). The participants were all chosen based on their propensity and perceived capacity to accurately respond to the questionnaire items. A consent letter was administered to them before they engaged in the research process.

### 3.2. Instruments

#### 3.2.1. Teacher Confirmation Scale

To measure this positive communication behavior, the “*Teacher Confirmation Scale (TCS)*” (Ellis, 2000) was employed. The scale entails 27 close-ended items ranging in their answers from 0 “Never” to 4 “Almost Always.” It is made of four different components, including “response to questions,” “demonstrated interest,” “teaching style,” and “absence of disconfirmation.” Examples of items from this inventory are: item (5) “*Checks on students’ understanding before going on to the next point*,” item (13) “*Takes time to answer students’ questions fully*,” and item (25) “*Incorporates exercises into lectures when appropriate*.” The Cronbach’s alpha reliability of 0.94 was found for this inventory.

#### 3.2.2. Perceived Caring Scale

To evaluate English learners’ standpoints concerning their instructors’ caring behaviors, the “*Perceived Caring Scale (PCS)*” (Teven and McCroskey, 1997) was administered to them. The PCS is comprised of 10 bipolar items, some of which are as follows: item (4) “*Unconcerned with me/Concerned with me*,” item (9) “*Understands how I feel/Does not understand how I feel*,” and item (10) “*Does not understand how I think/Understand how I think*.” The Cronbach’s alpha coefficient of PCS in this inquiry was 0.92.

#### 3.2.3. Learner empowerment questionnaire

The “*Learner Empowerment Questionnaire (LEQ)*” (Frymier et al., 1996) was distributed among participants to evaluate their sense of empowerment. It includes 30 items varying in their responses from 0 “Never” to 4 “Very often.” Some of these items are: item (1) “*I feel confident that I can adequately perform my duties*,” item (12) “*I like to talk about what I’m doing in my class with friends or family*,” and item (21) “*My success in this class is under my control*.” The LEQ’s reliability was estimated to be 0.97 in this research.

### 3.3. Data collection and analysis procedure

First, learners who signed and returned the consent forms were asked to respond to the electronic version of three pre-designed questionnaires. Then, some succinct explanations were offered to respondents regarding the completion of the questionnaires. Finally, the respondents (*N* = 391) completed the questionnaires and submitted them within 15 days. The respondents’ perceptions of the interplay between the constructs were statistically analyzed through the IBM SPSS Amos software. The IBM SPSS is an easy-to-use software that allows researchers and analysts “to specify, estimate, assess, and present models to show hypothesized relationships among variables” (Arbuckle, 2011, p. 1).

## 4. Results

Initially, confirmatory factor analysis (CFA) was run to check the validity of the constructs under investigation (i.e., teacher care, teacher confirmation, learner empowerment). The outcomes of this statistical procedure are presented below (Table 1).

**Table 1:** The outcomes of CFA.

			Estimate	S.E.	C.R.	*P*
Conscientiousness	←	Teacher care	0.999	0.113	11.743	0.000
Support	←	Teacher care	0.999	0.113	11.743	0.000
Inclusiveness	←	Teacher care	0.998	0.092	10.845	0.000
Meaningfulness	←	Learners’ empowerment	0.997			0.000
Competence	←	Learners’ empowerment	0.996	0.090	11.206	0.000
Impact	←	Learners’ empowerment	0.974	0.085	11.507	0.000
Responding to Questions	←	Teacher confirmation	0.995	0.082	12.358	0.000
Demonstrating Interest	←	Teacher confirmation	0.994	0.084	12.629	0.000
Interactive Teaching Style	←	Teacher confirmation	1.000			0.000
IC1	←	Inclusiveness	1.000			0.000
IC2	←	Inclusiveness	0.996	0.102	10.769	0.000
IC3	←	Inclusiveness	0.994	0.106	10.917	0.000
SU1	←	Support	1.000			0.000
SU2	←	Support	0.891	0.067	13.296	0.000
SU3	←	Support	0.823	0.068	12.136	0.000
SU4	←	Support	0.838	0.085	9.871	0.000
CN1	←	Conscientiousness	1.000			0.000
CN2	←	Conscientiousness	0.999	0.106	10.601	0.000
CN3	←	Conscientiousness	0.966	0.093	10.343	0.000
ME1	←	Meaningfulness	1.000			0.000
ME2	←	Meaningfulness	0.946	0.085	11.094	0.000
ME3	←	Meaningfulness	0.964	0.092	11.760	0.000
ME4	←	Meaningfulness	0.974	0.090	11.439	0.000
ME5	←	Meaningfulness	0.981	0.088	11.784	0.000
ME6	←	Meaningfulness	0.983	0.089	11.721	0.000
ME7	←	Meaningfulness	0.991	0.095	11.811	0.000
ME8	←	Meaningfulness	0.991	0.087	12.180	0.000
ME9	←	Meaningfulness	0.997	0.085	11.952	0.000
ME10	←	Meaningfulness	0.994	0.095	11.689	0.000
CM1	←	Competence	1.000			0.000
CM2	←	Competence	0.976	0.091	11.859	0.000
CM3	←	Competence	0.989	0.089	12.229	0.000
CM4	←	Competence	0.970	0.086	11.332	0.000
CM5	←	Competence	0.998	0.090	11.709	0.000
CM6	←	Competence	0.997	0.090	11.611	0.000
CM7	←	Competence	0.984	0.087	12.107	0.000
CM8	←	Competence	0.997	0.085	11.684	0.000
CM9	←	Competence	0.986	0.087	11.780	0.000
CM10	←	Competence	0.983	0.090	12.334	0.000
IM1	←	Impact	1.000			0.000
IM2	←	Impact	0.998	0.089	12.561	0.000
IM3	←	Impact	0.974	0.092	12.784	0.000
IM4	←	Impact	0.998	0.086	12.315	0.000
IM5	←	Impact	0.974	0.090	12.625	0.000
IM6	←	Impact	0.976	0.086	12.554	0.000
IM7	←	Impact	0.989	0.092	12.230	0.000
IM8	←	Impact	0.970	0.088	12.448	0.000
IM9	←	Impact	0.998	0.086	12.803	0.000
IM10	←	Impact	0.997	0.090	12.491	0.000
RQ5	←	Responding to	1.000			0.000
RQ4	←	Questions	0.986	0.078	12.615	0.000
RQ3	←	Responding to	0.975	0.079	13.334	0.000
RQ2	←	Questions	0.965	0.079	12.142	0.000
RQ1	←	Responding to	0.976	0.081	12.890	0.000
RQ6	←	Questions	0.989	0.081	13.734	0.000
RQ7	←	Responding to	0.970	0.085	12.757	0.000
RQ8	←	Questions	0.998	0.079	12.733	0.000
RQ9	←	Responding to	0.998	0.089	11.240	0.000
DI5	←	Demonstrating	1.000			0.000
DI4	←	Interest	0.976	0.073	13.691	0.000
DI3	←	Demonstrating	0.989	0.075	14.054	0.000
DI2	←	Interest	0.970	0.071	13.748	0.000
DI1	←	Demonstrating	0.998	0.075	13.589	0.000
DI6	←	Interest	0.982	0.075	13.161	0.000
DI7	←	Demonstrating	0.988	0.073	13.476	0.000
DI8	←	Interest	0.976	0.083	12.081	0.000
DI9	←	Demonstrating	0.989	0.071	13.355	0.000
IT5	←	Interactive	0.970			0.000
IT4	←	Teaching Style	1.068	0.086	12.474	0.000
IT3	←	Interactive	0.934	0.081	11.532	0.000
IT2	←	Teaching Style	0.941	0.078	11.997	0.000
IT1	←	Interactive	0.950	0.079	12.004	0.000
IT6	←	Teaching Style	0.976	0.084	13.152	0.000
IT7	←	Interactive	0.989	0.081	12.465	0.000
IT8	←	Teaching Style	0.970	0.085	12.349	0.000
IT9	←	Interactive	0.998	0.083	12.613	0.000

**Figure 1:**
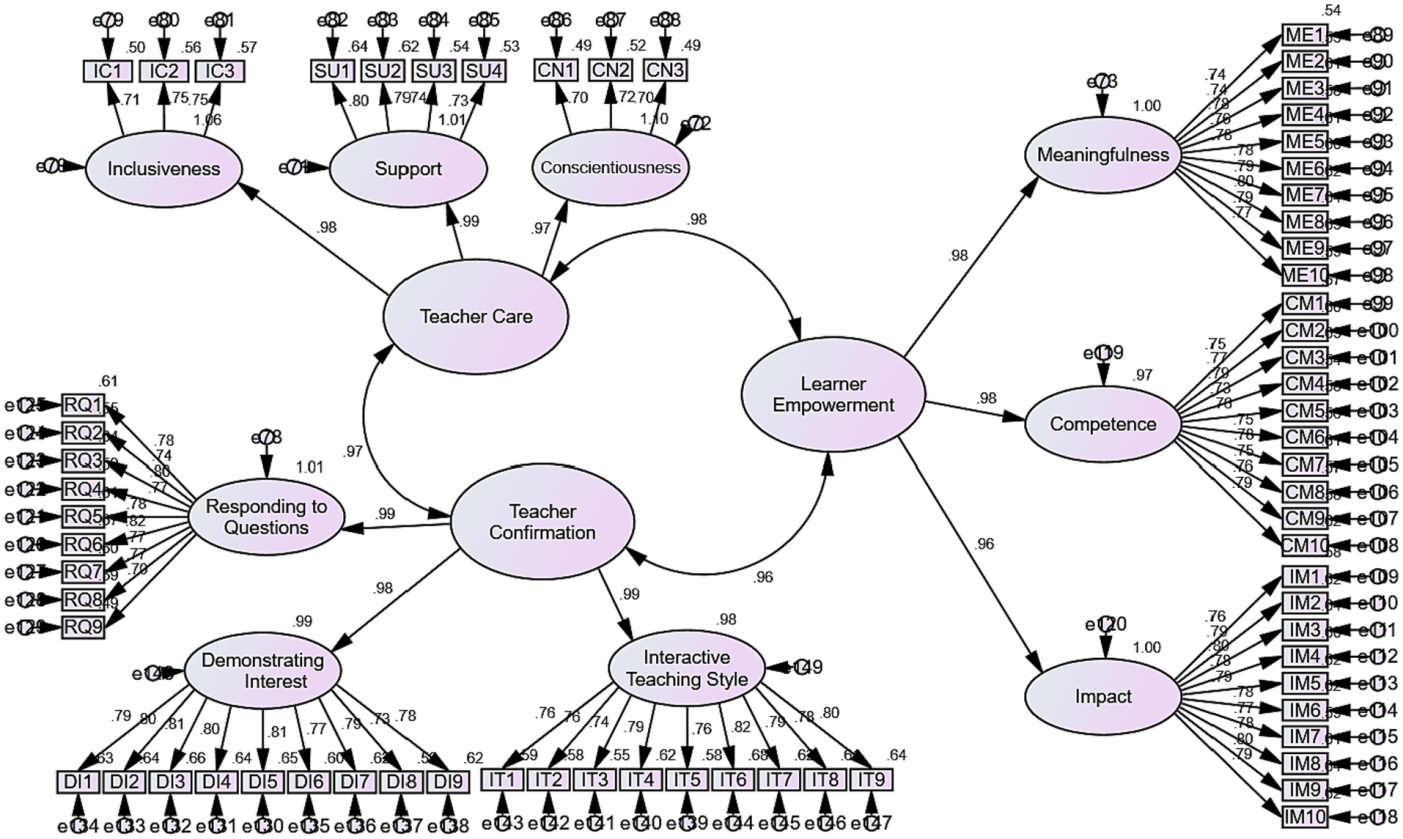
The initial CFA model.

The CFA outcomes disclosed that almost all of the values are higher than 0.5, acknowledging the validity of the variables and their components. With respect to the above values, the initial CFA model was drawn (Figure 1).

Next, the goodness of the initial CFA model was assessed and the results are demonstrated in the following table (Table 2).

**Table 2:** Evaluation of initial CFA model.

			Threshold		
Criteria		Terrible	Acceptable	Excellent	Evaluation
CMIN	4486.303				
DF	2132				
CMIN/DF	2.104	>5	>3	>1	Acceptable
RMSEA	0.072	>0.08	<0.08	<0.06	Acceptable
GFI	0.904	<0.9	>0.9	>0.95	Acceptable
CFI	0.934	<0.9	>0.9	>0.95	Acceptable
PNFI	0.900	<0.5	>0.5		Acceptable
TLI	0.928	>0.9	>0.9	>0.95	Acceptable

As represented in the above table, the fit indices, including “CMIN-DF,” “Goodness-of-Fit Index (GFI),” “Comparative Fit Index (CFI),” “Parsimonious Normed Fit Index (PNFI),” “Tucker–Lewis Index (TLI),” and “Root Mean Square Error of Approximation (RMSEA)” are all within specifications (Hu and Bentler, 1999). This confirms the quality of the initial CFA model. The discriminant validity of the model was then measured. Table 3 demonstrates the results of this measurement.

**Table 3:** Discriminant validity of the model.

	CR	AVE	MSV	MaxR(H)	Teacher confirmation	Learner empowerment	Teacher care
Teacher confirmation	0.996	0.993	0.996	0.985	0.998		
Learner empowerment	0.994	0.989	0.0994	0.964	0.994	0.997	
Teacher care	0.999	0.986	0.955	0.989	0.974	0.977	0.0998

The test results revealed that the proposed CFA model has an acceptable level of discriminant validity (the square root of AVE > inter-construct correlations). Following that, to respond to the first research question, the associations between teacher care, teacher confirmation, and learner empowerment were computed through a set of correlation tests. The results of correlation tests are displayed in the table below (Table 4).

**Table 4:** The interrelationships between teacher care, teacher confirmation, and learner empowerment.

			Estimate
Learner empowerment	↔	Teacher care	0.977
Teacher confirmation	↔	Learner empowerment	0.994
Teacher care	↔	Teacher confirmation	0.974

**Figure 2:**
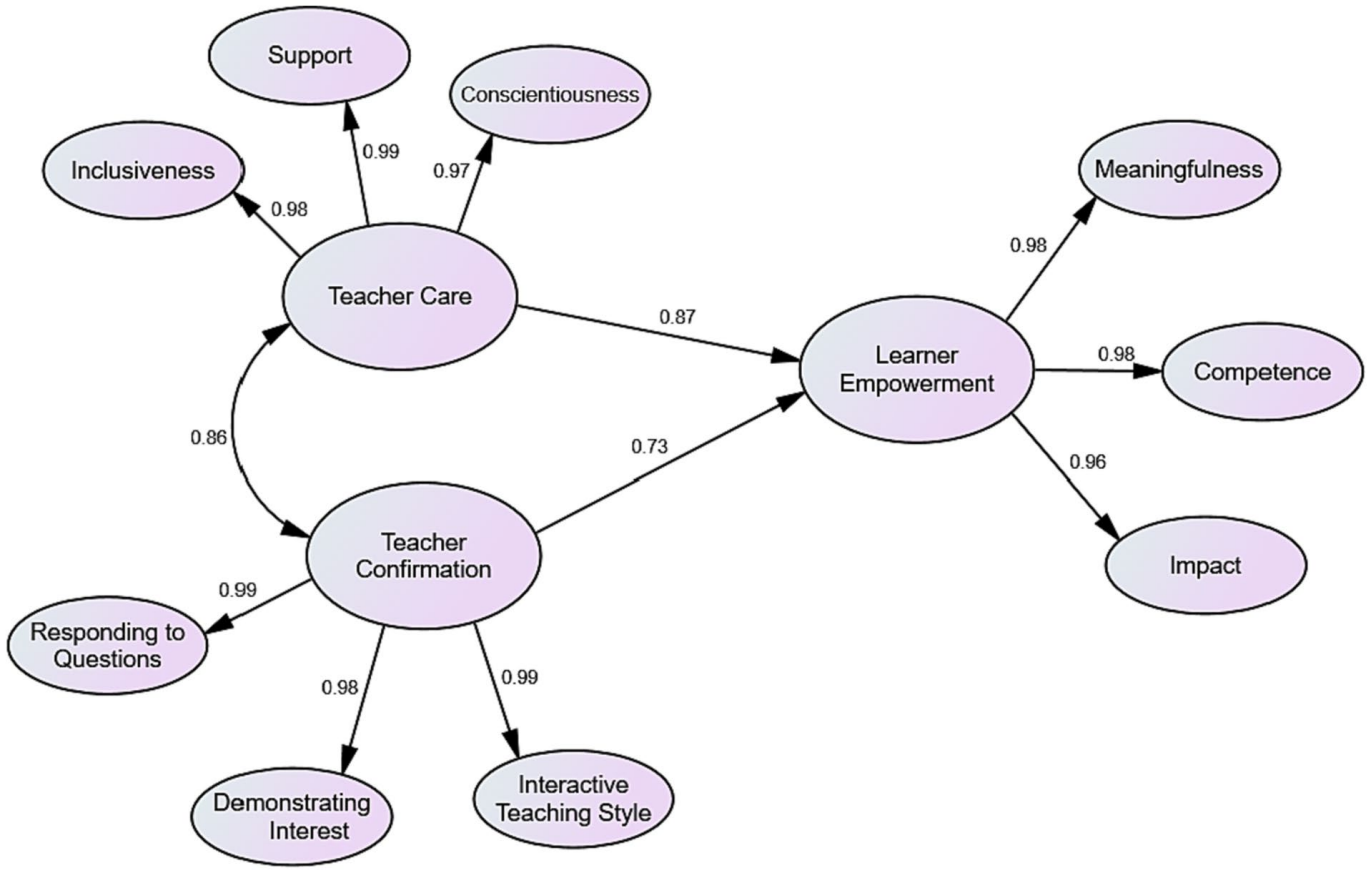
The linear regression model.

In accordance with the correlation test outcomes, a close and positive relationship exists between teacher care and learner empowerment. The test results also demonstrated that there is a favorable and strong association between teacher confirmation and learner empowerment. As the outcomes of the correlation test revealed, teacher confirmation is also closely related to teacher caring behaviors. Finally, to address the second research question, the dataset was subjected to linear regression analysis. The analysis findings are portrayed in the following figure (Figure 2).

According to the above figure, teacher care accounts for about 87 percent of the change in Chinese EFL learners’ empowerment, and teacher confirmation accounts for about 73 percent of the change. This means that these positive communicative behaviors can play a significant role in empowering EFL learners.

## 5. Discussion

This inquiry was planned to explore the interplay between teacher care, teacher confirmation, and learner empowerment in Chinese EFL classes. The research outcomes first revealed that teacher confirmation and teacher care are tightly connected to learner empowerment. The study findings also demonstrated that teacher confirmation and teacher care can bring significant changes in Chinese EFL learners’ empowerment. This means that positive communication behaviors like care and confirmation make EFL learners feel empowered.

The outcome of this inquiry about the close and favorable associations between teacher care, teacher confirmation, and learner empowerment lends support to the main principles of rhetorical/relational goal theory (Mottet et al., 2006; Wang et al., 2022; Wang and Derakhshan, 2023), which explained that positive communication behaviors help teachers promote learners’ educational behaviors like empowerment. This finding further accords with the idea of Myers et al. (2018), who asserted that satisfying learners’ relational and academic needs lead them to higher levels of empowerment. They maintained that teachers can meet their learners’ relational and academic needs only if they employ positive communicative behaviors such as care and confirmation. Furthermore, this also has echoed Houser and Hosek’s (2018) opinions, which suggested that positive communicative behaviors teachers use in instructional contexts strengthen their relationships with students. Houser and Hosek believed that strong teacher-student relationships can cause an increase in learners’ educational outcomes like empowerment. In addition, this result is aligned with that found by Derakhshan (2022), who discovered that teacher confirmation and teacher care are positively linked to learner empowerment. The outcome of the second research question on the role of teacher care and teacher confirmation in raising EFL learners’ empowerment is also consistent with the result of some earlier investigations. This outcome is in line with that obtained by Finn and Schrodt (2012), who reported that rhetorical and relational behaviors teachers employ in their classroom interactions can dramatically enhance their students’ empowerment. Moreover, this result is in tune with Diaz et al.’s (2016) findings, which divulged that positive communication behaviors can bring meaningful changes in learners’ sense of empowerment. Furthermore, this finding also verifies Derakhshan et al.’s (2022) results, which displayed that teacher caring and confirmation, as two positive interpersonal behaviors, play an important role in empowering EFL learners.

The findings of the current inquiry are subject to a set of limitations, which necessitate future empirical research on this issue. First and foremost, the current research study was conducted exclusively in China, as a result of which the findings might not be applicable to other EFL countries. Thus, future inquiries need to be performed in other EFL countries across the globe (Pan and Wang, 2023). Second, the present research only considered learners’ perceptions of the interrelationships between the variables under investigation. Therefore, it is recommended that the viewpoint of teachers regarding the interplay of these variables is examined in future inquiries. Third, in this inquiry, only self-report questionnaires were used to obtain the necessary information. To avoid the social desirability bias, future researchers are suggested to employ other data-gathering instruments as well. As has been indicated by Derakhshan et al. (2023), some emerging research methods should be employed to conduct research on emotional variables. Fourth, in the present inquiry, there was no control over the mediating role of participants’ demographic variables. Hence, future studies should inspect the influence of different demographic variables like gender, age, and academic degree on the interplay between teacher care, teacher confirmation, and learner empowerment.

## 6. Conclusion and pedagogical implications

This investigation was carried out with the aim of studying the implications of two positive communication behaviors, namely teacher care and teacher confirmation, for Chinese EFL learners’ empowerment. In fact, the present research sought to unravel the role of teacher care and teacher confirmation in empowering Chinese EFL learners. The results of linear regression analysis disclosed that teacher care and teacher confirmation play a significant role in empowering EFL learners. Put it another way, these teacher communication behaviors direct EFL learners to higher levels of empowerment. The results of this inquiry may have a number of theoretical and practical implications for L2 researchers, teacher educators, and language instructors. As to the theoretical implications, the present research extends the existing literature on the interplay between teacher communication behaviors and learner educational outcomes by highlighting the positive impacts of teacher care and teacher confirmation on English learners’ empowerment. As to the practical implications, the study outcomes may be of great help for language instructors in empowering their learners. In fact, the results of the present investigation expand language instructors’ understanding of the role of positive communication behaviors like care and confirmation in promoting learner empowerment. Likewise, these outcomes may also be insightful for teacher educators. According to the present results, positive communication behaviors have favorable impact on language learners’ empowerment. Thus, teacher educators are expected to teach their teacher students how to use positive communication behaviors in different instructional contexts.

## Data availability statement

The original contributions presented in the study are included in the article/supplementary material, further inquiries can be directed to the corresponding author.

## Ethics statement

The studies involving humans were approved by Academic Ethics Committee of Jiaozuo Normal College and Zhengzhou University. The studies were conducted in accordance with the local legislation and institutional requirements. The participants provided their written informed consent to participate in this study.

## Author contributions

ML: Formal analysis, Writing – original draft, Writing – review & editing. ZC: Data curation, Methodology, Visualization, Writing – review & editing.
